# Ultra-Processed Foods Consumption Is Associated With Intakes of Critical Nutrients Related to Non-Communicable Diseases Among Adults in Dakar, Senegal

**DOI:** 10.3389/ijph.2025.1608374

**Published:** 2025-05-21

**Authors:** Saliou Diombo Kébé, Adama Diouf, Papa Mamadou dit Doudou Sylla, Abdou Badiane, Olouwafemi Mistourath Mama, Nicole Idohou-Dossou

**Affiliations:** ^1^ Laboratoire de Recherche en Nutrition et Alimentation Humaine, Département de Biologie Animale, Université Cheikh Anta Diop, Dakar, Senegal; ^2^ Laboratoire des sciences Biologiques, Agronomiques, Alimentaires et de Modélisation des Systèmes, Université Gaston Berger, Saint-Louis, Senegal

**Keywords:** Ultra-processed foods consumption, critical nutrients, non-communicable diseases, adults, diet quality, Senegal

## Abstract

**Objectives:**

Nutritional transition in Senegal favors the exposure to ultra-processed foods (UPF) which are linked to the development of non-communicable diseases (NCDs). This study aimed to assess UPF consumption and their contribution to dietary intakes of critical nutrients associated with NCDs.

**Methods:**

Dietary intakes of 301 urban adults were assessed using a multi-step 24-hour dietary recall. Foods consumed were classified using the NOVA classification, and nutrient composition was determined using nutritional labels or food composition tables.

**Results:**

UPF contributed to 17.4% of total energy, 43% of free sugars, 26.9% of total fat, 24.4% of sodium and 24% of potassium intakes. Higher UPF consumption was associated with higher intakes of energy, free sugars, fat, potassium and protein. Higher UPF consumption was also positively associated with a non-recommended intake level of total fat (OR = 2.56; *p* = 0.002) while a negative association was found with non-recommended intake levels of potassium (OR = 0.01; *p* < 0.001) and protein (OR = 0.43; *p* = 0.009).

**Conclusion:**

UPF contribute significantly to the intakes of critical nutrients, are associated with *poor diet quality and* might be a major determinant of the inc*idence* and prevalence of *non-communicable diseases.*

## Introduction

Globalization, urbanization and significant demographic growth observed in most developing countries, such as Senegal, have resulted in a nutritional transition. This transition refers to the evolution and changes in dietary habits and the effects of these changes on nutrition and health [1, 2]. Dietary patterns characterized by high levels of fats, sugars, salt and energy-dense foods, but deficient in fiber and essential micronutrients, are becoming more common. This shift is associated with the rising prevalence of obesity, overweight and non-communicable diseases (NCDs), including cardiovascular disease (CVD), type 2 diabetes, stroke and certain cancers [3, 4]. According to the World Health Organization (WHO), NCDs are responsible for 74% of deaths worldwide [5] and unhealthy diets are a major cause, particularly due to excessive consumption of sugar, salt and fat [6]. These concerning nutrients are key components of ultra-processed foods (UPF), which are industrially produced with multiple ingredients and large quantities of free sugars, oils, and salt, as well as food additives designed to mimic the sensory qualities of natural foods [7, 8]. UPF are characterized by being ready-to-eat or requiring little preparation, facilitating consumption outside regular mealtimes and encouraging individual consumption [9]. In addition to their unbalanced and poor nutritional profile, UPF are hyper-palatable, addictive, have a long shelf life, economically accessible, and often subject to marketing strategies that promote overconsumption [8].

Recently, there has been increasing evidence that UPF consumption contributes to the development of obesity and NCDs such as type 2 diabetes, cardiovascular disease, and some types of cancer [10]. In addition, the proportion of UPF defines the global nutritional quality of the diet, and a high consumption of UPF is associated with lower variety and less nutritious diet. Indeed, they have higher energy density, and the increase in their proportion in the diet is generally associated with higher intakes of saturated fat, sodium and free sugars and lower intakes of fiber, protein, potassium and other essential vitamins and minerals [11, 12]. To prevent NCDs, the World Health Organization (WHO) has identified evidence-based recommendations including, reducing intakes of total and saturated fat, sodium and free sugars to limit weight gain, waist circumference and body fat percentage, prevent coronary heart disease, and reduce the risk of developing NCDs [13–16]. However, data have also shown an association between higher dietary fiber intake and a reduced risk of developing CVD, type 2 diabetes and some cancers [17]. Moreover, other studies have demonstrated that diets rich in protein are beneficial for the heart health [18] and that high potassium intake can improve blood pressure and reduce cardiovascular risk [19].

In Senegal, studies on food consumption focus mainly on food security issues, eating habits and dietary diversity, while industrial food consumption is not well covered. These studies are essentially based on qualitative data, sometimes non-representative, which does not provide an accurate measure of food consumption. They generally rely on indicators such as dietary diversity or food consumption frequency [20–23].

However, UPF are increasingly present in the Senegalese food environment as reported by a recent study which showed that of the more than 5,000 types of packaged products available on the market, over 70% are ultra-processed [24], but their association with the alarming prevalence of NCDs is unknown. Given these concerning trends, there is a need to investigate the consumption of UPF and their impact on the population’s dietary intake. To our knowledge, no comprehensive studies have yet been conducted in Senegal to assess the impact of UPF on the Senegalese diet. Therefore, this study aimed to assess UPF consumption and their contribution to dietary intakes of critical nutrients related to NCDs among urban adults in Dakar, Senegal.

## Methods

### Data Sources

This cross-sectional descriptive study included a convenience sample of 301 adults, all aged between 20 and 69 years, living in urban Dakar. The training for data collection was carried out by nutritionists during the week before starting the study. Data collectors were trained in the 24-hour recall methodology, including the use of food quantification tools and understanding the Nova classification. At the end of the training, a pre-test was conducted under realistic conditions to assess the data collectors’ skills. Data were collected in December 2021 using a two-stage sampling plan. First, a random selection was made in 25 urban census districts (CD), spread across nine (9) health districts in the Dakar medical region. Then, at least 12 households were randomly selected in each CD, using a sampling interval defined according to the size of the CD. Finally, one adult was randomly recruited from each household. People with special nutritional needs or any medical condition requiring a special diet were considered ineligible.

Food consumption was measured using the quantitative 24-hour dietary recall method, following the automated multiple-pass method [25]. This method allowed the step-by-step recording of all foods and beverages consumed on the previous day and the amounts of each. It consists of a continuous report of all foods and beverages consumed during the previous day. The participant was then asked, using a pre-established list of foods, about any foods that he might have omitted. Next, the person was asked to indicate the time and place of consumption, as well as the method of preparation, the quantities and any additions. At the end, a final check was performed, listing all the reported items. Given Senegalese eating habits, in which food consumption is a shared act, quantities of food were weighed directly or using non-standard measures (slice, unit, tablespoon, bag, handful, etc.) or substitutes (water, dried millet, modelling paste) to obtain the weight, which refers to the volume occupied by the quantity of food consumed. The 24-hour food recalls were performed once for each participant, on any randomly assigned day of the week, including weekends days, except for holidays or special occasions. A second recall was carried out on 20% of the sample, randomly selected to detect any significant changes in diet.

### Food Classification According to the NOVA System

All food items reported as consumed by the participants were categorized according to the NOVA classification [8]. The first step was to compile a list of all foods consumed by participants. Simple or single-ingredient foods and packaged products were included directly in the list. Multi-ingredient preparations were disaggregated, and their ingredients were obtained from the Senegalese standard recipe database before being included in the list. Each food or ingredient was assigned to one of the NOVA groups. Unprocessed or minimally processed foods, which are natural or have undergone minimal treatment to make them more edible, such as fresh fruit, milk, fish, etc. Processed culinary ingredients, which are food extracts from the first group or from nature through industrial processes such as pressing, extraction or refining, including vegetable oils, butter, sugar, etc. Processed foods, which are industrially manufactured by adding at least one ingredient from the second group (such as salt, sugar, oil or fat) to one or more foods from the first group, for example, bread, cheese or tinned fruit. Ultra-processed foods which are industrially manufactured and made up of several ingredients, food substances that are rarely or never used in cooking and cosmetic additives, such as soft drinks, reconstituted meat products, etc. [8]. Ultra-processed foods were then classified according to the 23 sub-categories in the Nova-UPF tool which had been adapted to the Senegalese context [26].

### Nutritional Intake Assessment

The real quantity of each food consumed was obtained by using the database of food conversion factors to convert estimates in non-standard measures or substitutes to the real weight of the food consumed in grams. Then, energy and nutritional intakes for total fat, saturated fat, free sugars, sodium, potassium, protein and dietary fiber were estimated using the 2019 West African Food Composition Table [27] and the French CIQUAL 2020 Nutritional Food Composition Table [28]. To estimate the dietary share of each category of the NOVA system, we calculated the total calories consumed by individuals, including the energy contribution of each NOVA group and subgroups of UPF, and therefore the corresponding proportion of calories from those. Average daily total energy intake (TEI), means critical nutrients intakes and the prevalence of non-recommended intakes of these nutrients, were estimated for the whole study population and by terciles of energy consumption from UPF. The following thresholds were used to identify individuals not meeting the WHO daily recommendations for the prevention of NCDs: ≥30% of TEI for total fat, ≥10% of TEI for saturated fat, ≥10% of TEI for free sugars, ≥2000 mg for sodium, <3,510 mg for potassium, <0.83 g per kg of body weight for protein and <25 g for dietary fiber [13–19].

### Statistical Analysis

Participants were divided into terciles, according to energy intake from UPF, to examine variation in the intake of critical nutrients and the prevalences of non-recommended intake levels for the prevention of NCDs. Each tercile represented one-third of the study population, ranging from participants with the lowest energy intake from UPF (first tercile) to those with the highest intake (third tercile). Student’s t-test, Pearson’s chi-square and Kruskal-Wallis test were used to evaluate differences between groups. Regression models were used to study associations between consumption of UPF, intakes of critical nutrients, energy intake and non-recommended intake levels across terciles of UPF energy contribution. All models were adjusted for the potential confounding factors including gender, age, age group, level of education and occupation. All data analyses were performed with the Stata^®^ 16.1 software and a significance level of p-value ≤0.05 was adopted.

## Results

The mean age of participants was 42 years, with 49.5% aged between 20 and 39 years. The distribution by sex showed a balance between women (49.8%) and men (50.2%). In terms of educational level, 28.6% of the participants had reached university and quarter of the participants had no occupation at the time of the study, while the majority were already working (65.1%) and the remainder were students (9.6%). Considering terciles of energy contribution from UPF, there was no significant difference in the distribution of participants based on socio-demographic characteristics (Table 1).

**TABLE 1:** Socio-demographic characteristics of participants across terciles of energy contribution of ultra-processed foods in a Senegalese urban adult population (N = 301) aged 20 years and older (Dakar, Senegal. 2021).

Characteristics	All (n = 301)	Terciles of energy intake from UPF			*P*
		T1	T2	T3	
Mean age, M ± SD	42 ± 14	44 ± 14	40 ± 14	41 ± 14	0.947
Gender, %(n)					
Women	49.8 (150)	49.5 (50)	53 (53)	47 (47)	0.695
Men	50.2 (151)	50.5 (51)	47 (47)	53 (53)	
Group age, %(n)					
20–39	49.5 (149)	41.6 (42)	54 (54)	53 (53)	0.322
40–59	34.2 (103)	39.6 (40)	29 (29)	34 (34)	
60–68	16.3 (49)	18.8 (19)	17 (17)	13 (13)	
Level of education, %(n)					
Below University	45.8 (138)	51.5 (52)	47 (47)	39 (39)	0.752
University	28.6 (86)	26.7 (27)	28 (28)	31 (31)	
Vocational school	16.3 (49)	13.8 (14)	16 (16)	19 (19)	
Others	9.3 (28)	7.9 (8)	9 (9)	11 (11)	
Occupation, %(n)					
No occupation	25.3 (76)	29.7 (30)	27 (27)	19 (19)	0.133
Student	9.6 (29)	6.9 (7)	14 (14)	8 (8)	
Worker	65.1 (196)	63.4 (64)	59 (59)	73 (73)	

**FIGURE 1:**
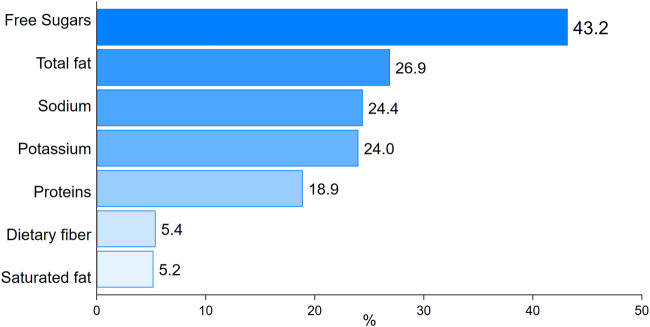
Relative contribution of ultra-processed foods to intake of critical nutrients.

Table 2 presents the TEI according to NOVA food groups and terciles of energy contribution from UPF. On average, the daily energy intake came mainly from unprocessed or minimally processed foods (1,005.9 ± 32.8 kcal; 56.9%), with the energy contribution of this category decreasing significantly with increasing UPF consumption (*p* < 0.001). Processed culinary ingredients accounted for an average of 353.5 ± 15.6 kcal, equivalent to 19.9% of TEI. Individuals in the highest tercile of UPF consumption had significantly lower intakes of processed culinary ingredients (*p* < 0.001). UPF were the third most important source of energy for our participants, with an average intake of 346.9 ± 25.8 kcal (17.4% of TEI), and the mean dietary contribution ranged from 2% to 38%, increasing significantly across terciles (*p* < 0.001). Processed foods contributed to an average of 91.2 ± 10.5 kcal, corresponding to 5.7% of TEI, and there was no statistical difference according to the level of consumption of UPF. Within ultra-processed products, the main energy sources were the sub-groups “Instant milk powder or instant chocolate powder” (7.2% of TEI), “Industrial mayonnaise ketchup or mustard” (3% of TEI), “Processed meats or meat spreads or nuggets” (1% of TEI), “Chocolate bars, confectionery or chewy products” (1% of TEI) and “Margarine” (1% of TEI). These combined ultra-processed sub-groups contributed twice as much as processed food overall. The contribution of ultra-processed products to intakes of critical nutrients is illustrated in Figure 1. A very important proportion of free sugars intake is provided by UPF (43%), and a contribution to more than a quarter of total fat (26.9%), sodium (24.4%), and potassium (24.0%). A considerable proportion of protein (18.9%) also comes from UPF, with lower percentages of dietary fiber (5.4%) and saturated fat (5.2%).

**TABLE 2:** Average and relative energy intake according to NOVA groups and ultra-processed food sub-groups in a Senegalese urban adult population (N = 301) aged 20 years and older (Dakar, Senegal. 2021).

NOVA groups	Energy		Terciles of energy contribution of UPF (%)			
	Mean ± SE (kcal)	% of TEI	T1	T2	T3	*P*
Unprocessed or minimally processed	1,005.9 ± 32.8	56.9	65.6	63.0	42.2	<0.001
Processed culinary ingredients	353.5 ± 15.6	19.9	25.6	19.4	14.8	<0.001
Processed foods	91.2 ± 10.5	5.7	6.8	5.3	5.0	0.863
Ultra-processed foods	346.9 ± 25.8	17.4	2.0	12.3	38.0	<0.001
Instant milk powder or instant chocolate powder	151.8 ± 19.7	7.2	0.3	5.1	16.1	<0.001
Industrial mayonnaise, ketchup or mustard	60.6 ± 9.2	3.0	0.4	1.3	7.2	0.026
Chocolate bars, confectionery or chewy products	22.6 ± 9.7	1.0	0.3	0.3	2.6	0.362
Processed meats or meat spreads or nuggets	17.7 ± 4.4	1.0	0.0	0.7	2.3	<0.001
Margarine	16.2 ± 2.8	1.0	0.3	1.1	1.5	0.001
Chocolate or hazelnut paste spreads	15.3 ± 3.8	0.9	<0.1	0.8	1.8	0.004
Soda, energizing or diet beverages	11.0 ± 2.2	0.6	0.1	0.9	0.8	0.025
Cookies or biscuits with or without fillings	9.7 ± 3.1	0.6	<0.1	0.2	1.4	0.013
Iced or flavored tea, coffee milk or flavored milk	10.4 ± 5.4	0.4	<0.1	0.0	1.1	0.026
Flavored drinks, concentrates and/or fruit nectars	6.6 ± 2.1	0.4	0.1	0.3	0.6	0.579
Flavored yoghurt or industrial curdled milk	6.3 ± 1.6	0.4	0.0	0.6	0.59	0.007
Frozen fries or frozen pizza	3.5 ± 1.4	0.2	<0.1	0.3	0.2	0.246
Chips, or any other type of packaged salty snack	3.1 ± 1.5	0.2	0.0	<0.1	0.5	0.060
Spreadable cheese	2.2 ± 0.8	0.2	0.1	0.2	0.2	0.558
Frozen ready-to-eat meal	3.0 ± 3.0	0.1	0.0	0.0	0.2	0.365
Bouillons, sauces, and industrial dressings	2.7 ± 0.2	0.1	0.2	0.1	<0.1	<0.001
Industrial cakes, muffins, or pastries	1.8 ± 1.6	0.1	0.0	0.0	0.2	0.133
Instant soup powder or instant noodles	1.1 ± 1.1	0.1	0.0	<0.1	0.0	0.365
Packaged industrial bread and rusks	0.9 ± 0.7	0.1	0.0	0.0	0.2	0.133
Sweetened breakfast cereals	0.4 ± 0.4	<0.1	0.0	0.0	0.1	0.365

TEI, Total Energy Intake; T1-T3, First, second and third terciles of energy contribution of UPF.

Table 3 indicates the average dietary intake of energy and critical nutrients. Participants in the highest tercile of energy contribution from UPF also consumed significantly more calories (1,995.8 ± 85.3 kcal; *p* = 0.001), had significantly higher intakes of total fat (78.7 ± 4.6 g; *p* = 0.000), free sugars (23.4 ± 3.1 g; *p* = 0.000), potassium (2,593.6 ± 203.3 g; *p* = 0.000), and protein (65.3 ± 3.8 g; *p* = 0.005) compared to those with lower energy intake from UPF. However, mean saturated fat intake was significantly lower at 4.8 ± 0.6 g (*p* = 0.024). Mean sodium and dietary fiber intakes were 2,560.7 ± 126.5 mg and 15.3 ± 0.8 g respectively, with no difference according to UPF consumption. Regression coefficients, both crude and adjusted, showed a positive association between increased proportion of UPF in the diet and dietary energy density (β = 355.6; *p* < 0.01), as well as intakes of total fat (β = 27.0; *p* < 0.001), free sugars (β = 17.8; *p* = < 0.001), potassium (β = 1,320.5; *p* < 0.001) and protein (β = 14.9; *p* < 0.01).

**TABLE 3:** Average dietary intakes of critical nutrients according to terciles of energy contribution of ultra-processed foods in an urban Senegalese adult population (N = 301) aged 20 years and older (Dakar, Senegal. 2021).

Nutrients	Overall diet (Mean ± SE)	Terciles of energy intake from UPF (Mean ± SE)				Coefficient of regression (*β*)	
		T1	T2	T3	*P*	Crude	Adjusted
Energy (kcal)	1797.6 ± 46.6	1,636.1 ± 9	1762.7 ± 61.7	1995.6 ± 85.3	0.001	359.5^a^	355.6^a^
Total fat (g)	61.9 ± 2.3	51.8 ± 3.6	55.5 ± 2.8	78.7 ± 4.6	<0.001	26.8^b^	27.0^b^
Saturated fat (g)	5.7 ± 0.3	6.4 ± 0.6	5.8 ± 0.5	4.8 ± 0.6	0.024	−1.6	−1.5
Free Sugars (g)	12.2 ± 1.2	5.5 ± 0.9	7.8 ± 0.6	23.4 ± 3.1	<0.001	17.9^b^	17.8^b^
Sodium (mg)	2,685.3 ± 89.5	2,709.8 ± 175.9	2,785.2 ± 158.8	2,560.7 ± 126.5	0.486	−149.1	−175.3
Potassium (mg)	1914.9 ± 85.6	1,366.1 ± 84.9	1790.5 ± 101.4	2,593.6 ± 203.3	<0.001	1,227.5^b^	1,320.5^b^
Proteins (g)	58.1 ± 1.9	50.8 ± 3.1	58.3 ± 2.7	65.3 ± 3.8	0.005	14.5^a^	14.9^a^
Dietary fibre (g)	15.1 ± 0.5	14.6 ± 0.8	15.3 ± 0.8	15.3 ± 0.8	0.749	0.6	0.4

^a^ p < 0.01.

^b^ p < 0.001.

The prevalence of non-recommended intake levels across terciles of dietary share of UPF are shown in Table 4. Overall, the highest prevalences of non-recommended intakes were found for potassium (90.7%), dietary fiber (90.4%), proteins (60.8%), total fat (49.2%) and sodium (33.5%). Non-recommended intake level of total fat intake increased significantly from 40.6% to 64% (*p* = 0.001) according to terciles of UPF consumption. However, there was a decreasing trend, with a lower prevalence of non-recommended intake levels in the upper tercile of UPF energy intake for saturated fat (1%; *p* = 0.042), potassium (78%; *p* = 0.01) and proteins (53%; *p* = 0.023). In addition, all individuals with non-recommended intake levels of free sugars were in the highest tercile, and there was no difference in dietary fiber intake. A strong positive association was found between increasing dietary share of UPF and non-recommended intake level of total fat (OR = 2.58; *p* = 0.002). Indeed, individuals in the highest tercile of UPF consumption had two times risk to exceed recommended intakes compared to the lowest tercile. In contrast, a negative association was found between terciles of energy intake from UPF and non-recommended intakes of potassium (OR = 0.01; *p* < 0.001) and protein (OR = 0.43; *p* = 0.009). Despite the elevated rate of non-recommended intake levels for potassium and protein, individuals in the highest tercile had extremely low probability of non-recommended potassium intakes and 57% less chance of non-recommended protein intakes compared to those in the lowest tercile.

**TABLE 4:** Prevalence (%) of non-recommended nutrient intake levels according to terciles of dietary share of ultra-processed foods in an urban Senegalese adult population (N = 301) aged 20 years and older (Dakar, Senegal. 2021).

Nutrients (thresholds)	Overall	Terciles of energy intake from UPF				OR adjusted	*P*
		T1	T2	T3	*P*		
Total fat (>30% of TEI)	49.2	40.6	43.0	64.0	0.001	2.58	0.002
Saturated fat (>10% of TEI)	2.7	5.9	1.0	1.0	0.042	0.06	0.052
Free Sugars (≥10% of TEI)	2.3	0.0	0.0	7.0	0.001	-	-
Sodium (≥2g)	33.5	32.7	30.0	38.0	0.475	1.29	0.420
Potassium (<3510 mg)	90.7	99.0	95.0	78.0	0.000	0.01	0.000
Proteins (<0.83/Kg)	60.8	71.3	58.0	53.0	0.023	0.43	0.009
Dietary fibre (<25g)	90.4	92.1	94.0	85.0	0.076	0.40	0.072

## Discussion

In this cross-sectional study conducted in a population of Senegalese adults, surveyed in 2021, we found that unprocessed or minimally processed foods contribute the most to calorie intake with 56.9%, while UPF provide 17.4% of daily TEI. This proportion of dietary energy from UPF is the same (17%) as that reported for a group of Indian adults [29] but lower than those found among other adult populations in South Africa [30], Switzerland [31], Mexico [32] and the USA [33]. These are all countries with higher incomes and eating habits that are very different from our context. The difference in proportions between unprocessed or minimally processed foods and UPF reflects a diet still heavily based on natural or minimally processed foods, which are staple foods. Processed culinary ingredients also account for a significant proportion (19.9%) due to culinary habits that involve their use in cooking or as flavor enhancers, additions or accompaniments to meals. The share of UPF also reflects the dynamic of the current nutritional transition, with cereals such as rice, wheat and maize used to account for up to 70% of caloric intake in low-income countries [34]. This proportion of minimally processed products is therefore being reduced in favor of UPF, which are becoming increasingly important in the diets of populations in developing countries.

The UPF subcategory that contributed the most to energy intake was “Instant milk powder or instant chocolate powder” unlike in other adult populations in Switzerland, Mexico, United States [31–33] and in a study in Brazil [11], where most of the ultra-processed calories came from bakery, confectionery, and other similar products. Regarding Senegalese dietary habits, this is mainly due to instant powdered milk, which occupies a very important place in the Senegalese diet, as it is widely consumed with couscous of millet, used for breakfast and in a large variety of daily preparations. It’s made from skimmed milk and vegetable fat with emulsifiers and anti-caking agents. Non-industrial breads and pastries are most commonly consumed, while highly processed breads, pastries, biscuits or cakes are less commonly consumed by adults. Salty sauces (mayonnaise, mustard and ketchup), which have all become very popular, also contributed significantly to calorie intake from UPF, due to their widespread use as accompaniments to the consumption of traditional dishes, fast food or sandwiches.

UPF mainly provides the three most critical nutrients, accounting for almost half of the intake of free sugars (43.2%), around a quarter of total fat (26.9%) and sodium (24.4%). However, they do not have a major influence on dietary fiber and saturated fat intakes. Sweetened ultra-processed products are therefore the most consumed type in our study population. The percentage of free sugars provided by UPF is higher than that reported in other studies in India, Switzerland and Australia [29, 31, 35], while the percentage of protein intake in these studies is similar to that reported in our population (18.9%). The share of total fat provided by UPF is equivalent to that reported in a Brazilian study [11], but sodium intake from UPF is lower compared to that of the Indian adult population [29]. These variations can be explained by the differences in diets, availability of UPF and local public health policies towards ultra-processed products.

The associations found between increased consumption of UPF and a significant increase in energy, free sugars and total fat intakes were also reported by several studies [11, 32, 35–38]. In addition to the processing they undergo, the nutritional composition of UPF includes high levels of sugars and fats, conferring on them a high energy density. However, our results also showed a positive association between UPF consumption and both potassium and protein intakes, and a significant improvement in compliance with WHO recommendations for both, which is contrary to the results reported by other studies [11, 32, 35–38]. Indeed, UPF contributed significantly to potassium (24%) and protein (18.9%) intakes. Although unprocessed or minimally processed foods are the most commonly consumed type of food, these are mainly staple foods such as cereals and starches, while dark green leafy vegetables, pulses, nuts or seeds, and fruits, which provide better potassium intake, are the food groups least consumed by adults in Dakar [39]. Moreover, potassium chloride is used in UPF as a substitute for sodium chloride and for improving the taste of certain products [40], particularly in processed meat and processed dairy products, which are well-consumed by our study population. All this would explain why a good proportion of dietary potassium is provided by UPF and reinforces the hypothesis that the use of potassium (potassium chloride) in industrial foods could strengthen compliance with potassium intake recommendations [41]. A similar observation applies to protein intake, as adults in developing countries do not consume enough minimally processed animal products. Instant milk powder, which contains significant amounts of protein, is the most consumed UPF subgroup, providing more energy than all the category of processed foods. Ultra-processed meat products (sausages, salami, corned beef, etc.) are also widely consumed and very accessible. This would explain why UPF also significantly contributes to our population’s protein intake. Greater consumption of UPF is also associated with higher non-recommended intake of total fat, which corroborates the results of a study conducted in Australia [35]. It was also found that only 7% of individuals, all in the highest tercile of energy intake from UPF, had intakes of free sugars that exceeded the WHO recommendation. This is of course due to the much higher intakes of those who consume more UPF.

The overall findings suggest that the ultra-processed products consumed by our study population are mainly characterized by their high fat and sugar content, which has a significant impact on the intake levels of our subjects. UPF are also a potential source of improved protein and potassium intakes, although not sufficient to reach recommended levels. As already shown in other contexts [11, 42, 43], this study confirms that UPF consumption is a key factor in determining the nutritional quality of diets. Given the established link between UPF consumption and NCDs, the proportion of UPF consumption in our diet is not negligible. Moreover, with the agri-food sector, under the influence of multinational companies that are aggressively expanding into African markets, favoring the growing presence of UPF, which are affordable, convenient and highly marketed, particularly in urban areas [20, 44]. There is an urgent need for public policymakers in low-income countries such as Senegal, to address the public health issue of UPF. This will make it possible to establish a regulatory framework for the marketing and promotion of these products, as well as initiatives aimed at raising awareness and limiting the consumption of unhealthy foods in order to promote a better food environment for the population.

This study is the first, to our knowledge, to address the contribution of ultra-processed foods to nutrients of concern intakes and their impact on diet quality in West Africa. The NOVA classification, which has highlighted ultra-processed products and their association with NCDs was used. In this study, the Nova-UPF tool adapted to the Senegalese context [26, 45], which identifies the different sub-categories of UPF available in Senegal, allowed us to assess in detail and accurately which types of ultra-processed products were most consumed. This study is an important starting point on which further larger-scale studies can be designed.

However, study’s limitations may be its small sample size, as it is limited to a single population group and is not of national scope. There are also the biases inherent to the 24-hour recall methodology, including memory effects, intra-individual variability, and reporting errors, which were minimized by performing a second recall in a subsample and implementing rigorous standardization of the data collection protocol.
